# Cetuximab-Mediated Protection from Hypoxia- Induced Cell Death: Implications for Therapy Sequence in Colorectal Cancer

**DOI:** 10.3390/cancers12103050

**Published:** 2020-10-20

**Authors:** Hans Urban, Gabriele D. Maurer, Anna-Luisa Luger, Nadja I. Lorenz, Benedikt Sauer, Christopher Stroh, Jörg Trojan, Michel Mittelbronn, Joachim P. Steinbach, Patrick N. Harter, Michael W. Ronellenfitsch

**Affiliations:** 1 Dr. Senckenberg Institute of Neurooncology, University Hospital Frankfurt, Goethe University, 60528 Frankfurt am Main, Germany; hans.urban@kgu.de (H.U.); gabriele.maurer@kgu.de (G.D.M.); anna-luisa.luger@kgu.de (A.-L.L.); nadja.lorenz@kgu.de (N.I.L.); benedikt.sauer@kgu.de (B.S.); joachim.steinbach@kgu.de (J.P.S.); 2 University Cancer Center Frankfurt (UCT), University Hospital Frankfurt, Goethe University, 60590 Frankfurt am Main, Germany; patrick.harter@kgu.de; 3 German Cancer Consortium (DKTK), Partner Site Frankfurt/Mainz, 60590 Frankfurt am Main, Germany; 4 Merck Healthcare KGaA, 64289 Darmstadt, Germany; christopher.stroh@merckgroup.com; 5 Department of Gastroenterology, University Liver and Cancer Centre, Frankfurt University Hospital, Goethe University, 60590 Frankfurt am Main, Germany; trojan@em.uni-frankfurt.de; 6 Luxembourg Center of Neuropathology (LCNP), 3555 Dudelange, Luxembourg; michel.mittelbronn@lns.etat.lu; 7 Luxembourg Centre for Systems Biomedicine (LCSB), University of Luxembourg, 4367 Belvaux, Luxembourg; 8 Department of Oncology (DONC), Luxembourg Institute of Health (LIH), 1526 Luxembourg, Luxembourg; 9 National Center of Pathology (NCP), Department of Anatomic and Molecular Pathology, 3555 Dudelange, Luxembourg; 10 Frankfurt Cancer Institute (FCI), University Hospital Frankfurt, Goethe University, 60596 Frankfurt am Main, Germany; 11 Institute of Neurology (Edinger-Institute), University Hospital Frankfurt, Goethe University, 60528 Frankfurt am Main, Germany

**Keywords:** colon carcinoma, cetuximab-bevacizumab therapy sequence, epidermal growth factor receptor, vascular endothelial growth factor receptor, hypoxia

## Abstract

**Simple Summary:**

Therapeutic antibodies are an integral part of treatment regimens for metastasized colorectal cancer. In KRAS wildtype tumors both bevacizumab and cetuximab are active. While bevacizumab has previously been shown to induce tumor hypoxia, we here report that EGFR inhibition by cetuximab protects colon cancer cells from hypoxia-induced cell death. This effect appears to be responsible for the inferior efficacy of a treatment sequence of bevacizumab followed by cetuximab versus an inverse sequence that we observed in a colorectal cancer mouse model. It also offers a mechanistic explanation for effects observed in clinical trials such as underadditive or even detrimental effects when combining bevacizumab and cetuximab (CAIRO2 trial) and the superior efficacy of first line cetuximab (FIRE-3 trial) under chemotherapy backbones in colorectal cancer.

**Abstract:**

Monoclonal antibodies like cetuximab, targeting the epidermal growth factor receptor (EGFR), and bevacizumab, targeting the vascular endothelial growth factor (VEGF), are an integral part of treatment regimens for metastasized colorectal cancer. However, inhibition of the EGFR has been shown to protect human glioma cells from cell death under hypoxic conditions. In colon carcinoma cells, the consequences of EGFR blockade in hypoxia (e.g., induced by bevacizumab) have not been evaluated yet. LIM1215 and SW948 colon carcinoma and LNT-229 glioblastoma cells were treated with cetuximab, PD153035, and erlotinib and analyzed for cell density and viability. The sequential administration of either cetuximab followed by bevacizumab (CET->BEV) or bevacizumab followed by cetuximab (BEV->CET) was investigated in a LIM1215 (KRAS wildtype) and SW948 (KRAS mutant) xenograft mouse model. In vitro, cetuximab protected from hypoxia. In the LIM1215 model, a survival benefit with cetuximab and bevacizumab monotherapy was observed, but only the sequence CET->BEV showed an additional benefit. This effect was confirmed in the SW948 model. Our observations support the hypothesis that bevacizumab modulates the tumor microenvironment (e.g., by inducing hypoxia) where cetuximab could trigger protective effects when administered later on. The sequence CET->BEV therefore seems to be superior as possible mutual adverse effects are bypassed.

## 1. Introduction

Colorectal cancer (CRC) is the second most common cancer in women and the third in men worldwide [1]. Approximately 20% of patients initially present with metastatic disease [2]; despite therapeutic advances in the last decades, their prognosis remains poor. Combined with 5-fluorouracil (5-FU)-based chemotherapy regimens, monoclonal antibodies have become an integral part of treatment for metastasized colorectal cancer (mCRC) [3]. These biological drugs inhibit either the vascular endothelial growth factor A (VEGF-A) (i.e., bevacizumab), or the epidermal growth factor receptor (EGFR) (i.e., cetuximab and panitumumab), and can be chosen depending on *RAS* mutation status [4,5] or primary tumor site [6]. While bevacizumab is active in both *RAS* mutant and *RAS* wildtype tumors, EGFR antibodies are not recommended for the treatment of *RAS* mutant CRC. Although both anti-VEGF and anti-EGFR agents are active in this setting, the addition of panitumumab [7] or cetuximab [8] to bevacizumab plus standard chemotherapy resulted in shorter progression-free survival (PFS), major toxicity, and inferior quality of life in *RAS* status unselected patient cohorts in the PACCE (NCT00115765) and CAIRO2 (NCT00208546) trials. Subgroup analyses revealed a decreased overall survival (OS) of patients with *KRAS* mutant tumors who had received cetuximab and bevacizumab [8]. In patients treated with an oxaliplatin-based regimen, a trend toward a shorter OS was observed even in the *KRAS* wildtype cohort when panitumumab was administered in addition to bevacizumab [7]. Thus, therapy with either bevacizumab or an anti-EGFR antibody plus chemotherapy is regarded as the current standard of first-line therapy in mCRC patients. Upon progression of disease, switching from one antibody regimen to another is a frequently used strategy. Unfortunately, limited data exist on the impact of the sequence of therapies. The FIRE-3 trial aspired to compare cetuximab with bevacizumab in addition to first-line treatment with 5-FU, folinic acid, and irinotecan (FOLFIRI) in patients without tumor *KRAS* exon 2 mutations [5]. Although response and PFS rates did not differ significantly between treatment groups, the FOLFIRI plus cetuximab combination was associated with a longer OS. Further analysis suggested that the efficacy of second-line therapy was responsible for the difference in OS, with the sequence of cetuximab before bevacizumab (CET->BEV) being superior to bevacizumab before cetuximab (BEV->CET) [5,9]. Similar observations were made in an exploratory analysis including patients from PEAK (NCT00819780), PRIME (NCT00364013), and Study 181 (NCT00339183) trials who received either first-line panitumumab or first-line bevacizumab, followed by second-line inhibition of VEGF or EGFR, respectively [10]. The fact that adding one drug impairs the efficacy of another implies either pharmacokinetic interactions such as a reduced drug exposure or a biological effect. So far, there is no evidence for drug–drug interactions and little evidence for a reduced exposure to cetuximab after administration of bevacizumab [11,12]. Regarding biological mechanisms, even transient antiangiogenic therapy induced sustained hypoxia and other changes in the tumor microenvironment [13,14,15], and induction treatment with anti-VEGF therapy has been described in association with resistance to cetuximab [11,12]. These findings may constitute a rationale for a second-line treatment with bevacizumab. In glioma cells, pharmacological EGFR blockade confers protection from hypoxia-induced cell death [16], and starvation conditions attenuated the cytotoxic effect of EGFR inhibition [17]. Therefore, we hypothesized that chronic hypoxia induced by first-line bevacizumab therapy could antagonize the efficacy of second-line EGFR-antibodies in mCRC. In order to interrogate this hypothesis, we first conducted in vitro experiments using the cetuximab-sensitive colon cancer cell lines LIM1215 and SW948 [18]. Subsequently, we sought to identify the potential effects of therapy sequence on tumor growth in subcutaneous in vivo tumor models.

## 2. Results

### 2.1. Cetuximab Does Not Influence Cell Growth or Viability in Normoxia

**Figure 1:**
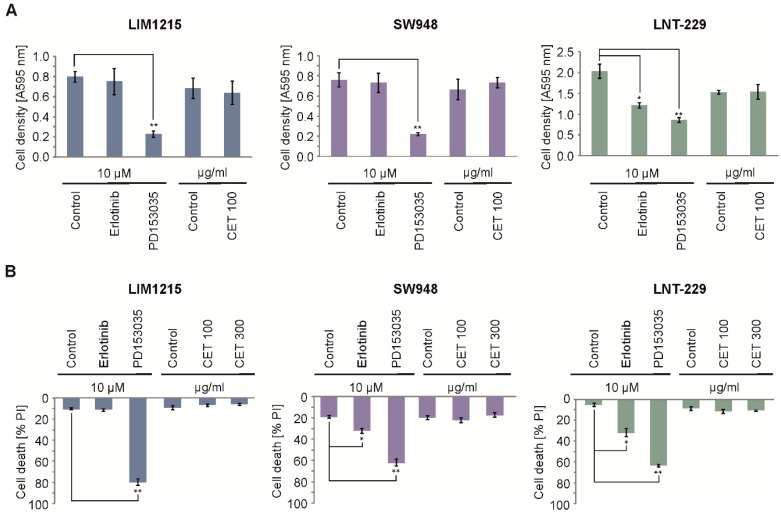
Growth inhibition and toxicity profile of EGFR inhibitors. (**A**) LIM1215, SW948, and LNT-229 cells were incubated with vehicle, 10 µM erlotinib, 10 µM PD153035, or 100 µg/mL cetuximab (CET 100) under normoxia for 48 h in serum-free medium. Cell density was assessed by crystal violet (CV) staining (*n* = 3, mean ± SD, * *p* < 0.05, ** *p* < 0.01). (**B**) LIM1215, SW948, and LNT-229 cells were incubated as in (**A**) with an additional 300 µg/mL cetuximab (CET 300) condition. Cell death was measured by propidium iodide (PI) uptake (*n* = 3, mean ± SD, * *p* < 0.05, ** *p* < 0.01).

To investigate the effects of EGFR inhibition in colon carcinoma cells, LIM1215 and SW948 cells were exposed to PD153035 and cetuximab under normoxic conditions. Both substances inhibited EGFR downstream signal transduction to a similar degree (Figure S1). However, EGFR inhibition with PD153035 triggered cell death in both LIM1215 and SW948 cells, while cetuximab did not induce cytotoxic effects (Figure 1A,B).

### 2.2. Cetuximab Protects Colon Carcinoma Cells From Hypoxia-Induced Cell Death

**Figure 2:**
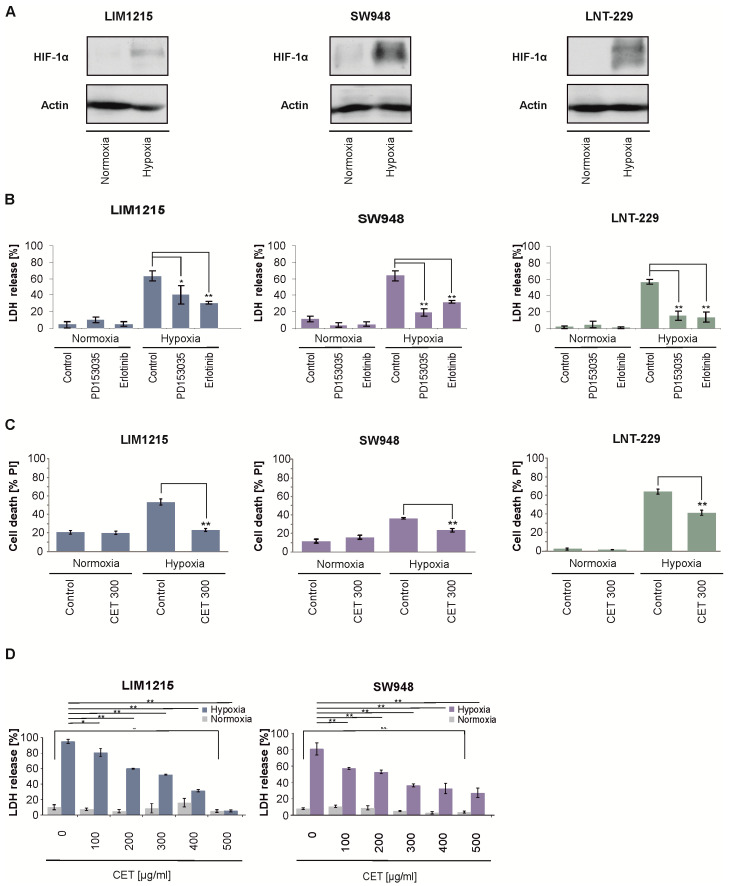
EGFR inhibition protects colon carcinoma and glioma cells from hypoxia-induced cell death. LIM1215, SW948, and LNT-229 cells were incubated in serum-free medium containing 2 mM glucose under normoxic or hypoxic conditions. (**A**) Induction of hypoxia-inducible factor HIF-1α in hypoxia was verified by immunoblot, the uncropped immunoblot figure in Figure S7. (**B**) Cells were treated with 10 µM erlotinib, 10 µM PD153035, and vehicle, respectively. Cytotoxicity was estimated by LDH release (*n* = 4, mean ± SD, * *p* < 0.05, ** *p* < 0.01). (**C**) Cells were incubated with vehicle or 300 µg/mL cetuximab (CET 300). Cell death was quantified by PI uptake (*n* = 3, mean ± SD, ** *p* < 0.01). (**D**) LIM1215 and SW948 cells were treated with vehicle or cetuximab (CET) at concentrations ranging from 100 to 500 µg/mL. Cell death was quantified by LDH release (*n* = 4, mean ± SD, ** *p* < 0.01, n.s., not significant).

We have previously shown that EGFR inhibition by PD153035 and erlotinib protects glioma cells from hypoxia-induced cell death [16,19]. First, we examined whether the treatment induced hypoxic stress in the cells and detected this by an increase in hypoxia-inducible factor HIF-1α (Figure 2A). Similarly, EGFR inhibition with PD153035 and erlotinib reduced the cell death of colon carcinoma cells under hypoxic conditions compared to the vehicle control (Figure 2B). Cetuximab reduced hypoxia-induced cell death in all tested cell lines in a comparable manner (Figure 2C). This effect was dose-dependent and observed at concentrations that have been measured in human serum after administration of standard treatment doses (Figure 2D and Figure S2) [20].

### 2.3. EGFR Gene Suppression Impairs Cell Growth and Protects From Hypoxia-Induced Cell Death

**Figure 3:**
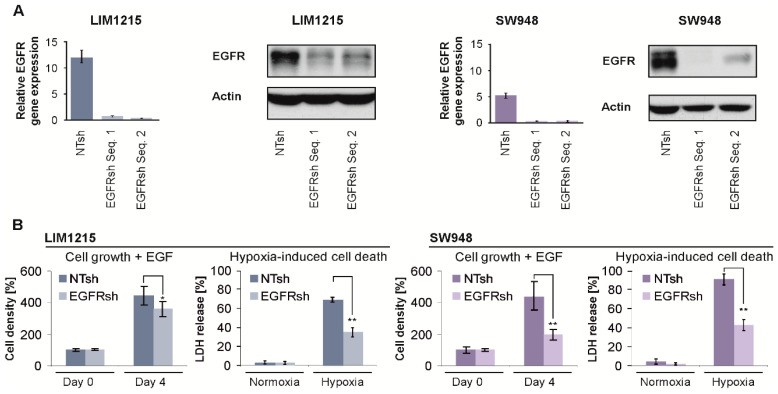
Generation and characterization of *EGFR* gene-suppressed colon carcinoma cells. (**A**) LIM1215 and SW948 cells were transduced with a lentivirus to express a non-targeting control shRNA (NTsh) or either of two shRNA sequences targeting *EGFR* (EGFRsh Seq. 1 and 2). Gene suppression was verified by qPCR (left, *n* = 3, mean ± SD) and immunoblot (right), the uncropped immunoblot figure in Figure S7. (**B**) LIM1215 and SW948 NTsh and EGFRsh (Seq. 1) cells were grown in serum-free medium supplemented with 10 ng/mL epidermal growth factor (EGF) for four days. Cell density was quantified by CV staining (left, relative to day 0, *n* = 5, mean ± SD, * *p* < 0.05, ** *p* < 0.01). In parallel, cells were incubated in serum-free medium containing 2 mM glucose under normoxic or hypoxic conditions. Cell death was measured by LDH release (right, *n* = 4, mean ± SD, ** *p* < 0.01).

To rule out any off-target effects of EGFR inhibitors, we generated *EGFR* gene-suppressed cells. Quantitative PCR and immunoblot confirmed stable gene suppression of *EGFR* in LIM1215 and SW948 cells (EGFRsh) compared to the control cells (NTsh) (Figure 3A). To evaluate a functional consequence of the reduced *EGFR* expression, we exposed cells to EGF. After four days, the cell density of the EGFRsh cells was lower than that of the NTsh cells (Figure 3B, left). Similar to EGFR inhibitors, *EGFR* gene suppression protected cells from hypoxia-induced cell death (Figure 3B, right, and Figure S3A) and cetuximab no longer conferred protection from hypoxia-induced cell death in EGFRsh cells (Figure S3B). Bevacizumab did not affect hypoxia-induced cell death in both LIM1215 and SW948 NTsh and EGFRsh cells (Figure S3C). Induction of HIF-1α and its target BNIP3 was also detectable in LNT-229 glioma cells (Figure S3D) as well as in LIM1215 and SW948 cells in the presence of cetuximab and bevacizumab in hypoxia (Figure S3E).

### 2.4. The Sequence of Cetuximab and Bevacizumab Administration Influences Outcome in a LIM1215 and a SW948 Subcutaneous Mouse Model

**Figure 4:**
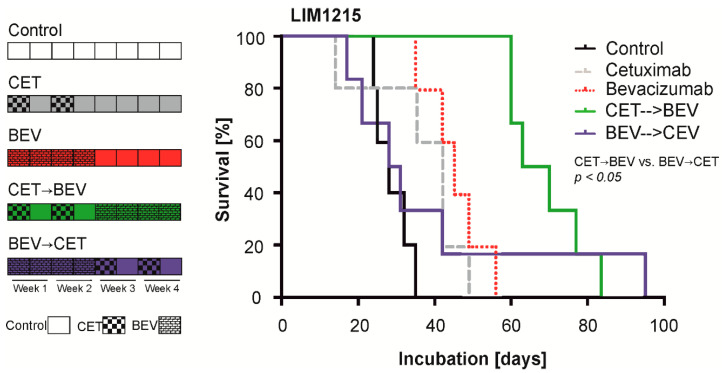
Cetuximab and bevacizumab in mono- or sequential therapy of subcutaneous LIM1215 tumors. Mice were treated either with PBS (control) for four weeks (two injections per week), with cetuximab (CET) for two weeks (one injection of CET 30 µg/g body weight and one injection of PBS per week), followed by PBS for two weeks (two injections per week), with bevacizumab (BEV) for two weeks (two injections of BEV 10 µg/g body weight per week), followed by PBS for two weeks (two injections per week), with CET for two weeks followed by BEV for two weeks (CET->BEV) or with BEV for two weeks followed by CET for two weeks (BEV->CET). Tumor volume was measured at the indicated intervals (control, CET, BEV: *n* = 5; CET->BEV, BEV-> CET: *n* = 6).

We hypothesized that the effect of bevacizumab-mediated tumor hypoxia could be counteracted by EGFR inhibition, partially explaining the detrimental effects when combining both antibodies in clinical trials. Furthermore, we were interested in which antibody should be used first to exploit the full potential of the drugs. We therefore treated subcutaneous LIM1215 tumors with a monotherapy consisting of either cetuximab or bevacizumab or a sequential combination therapy. The cetuximab-bevacizumab (CET->BEV) sequence prolonged survival to a greater extent (median 66.5 days, range 60 to 83 days) than the inverse sequence (BEV->CET, median 29 days, range 17 to 95 days, *p* = 0.043, Figure 4). Monotherapy with cetuximab (CET, median 42 days, range 14 to 49 days) or bevacizumab (BEV, median 45 days, range 35 to 56 days) was similarly active and extended survival in comparison to placebo (median 28 days, range 24 to 35 days, Figure 4; Figure S4).

**Figure 5:**
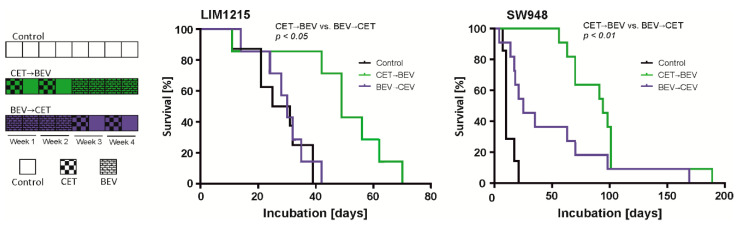
Impact of different treatment sequences on subcutaneous LIM1215 and SW948 tumor growth. Mice were treated either with PBS (control) for four weeks (two injections per week), with CET for two weeks followed by BEV for two weeks (CET->BEV) or with BEV for two weeks followed by CET for two weeks (BEV->CET). Tumor volume was measured at the indicated intervals (Lim1215 control: *n* = 8; CET->BEV, BEV->CET: *n* = 7; SW948 control: *n* = 7; CET->BEV, BEV->CET: *n* = 11). Growth data of each individual tumor are given in Figure S4.

To investigate whether this effect was also detectable in *KRAS* mutant colon carcinoma cells, we repeated the experiment with LIM1215 and SW948 cells in a slightly modified form (Figure 5). Here, only the sequence therapy groups and the control group were examined. This study was powered to compare the sequence therapies with each other. Again, the mice in the CET->BEV group survived longer than those in the BEV->CET group (Figure 5, Figure S4). This effect was also present in *KRAS* mutant SW948 cells (LIM1215 CET->BEV: median 49 days, range 11 to 70 days, BEV->CET: median 30 days, range 14 to 42 days, *p* = 0.022, SW948 CET->BEV: median 94 days, range 56 to 172 days, BEV->CET: median 25 days, range 4 to 169 days, *p* = 0.007).

### 2.5. Subcutaneous Tumor Xenografts Exhibit Larger Areas of Necrosis When Treated With Bevacizumab

**Figure 6:**
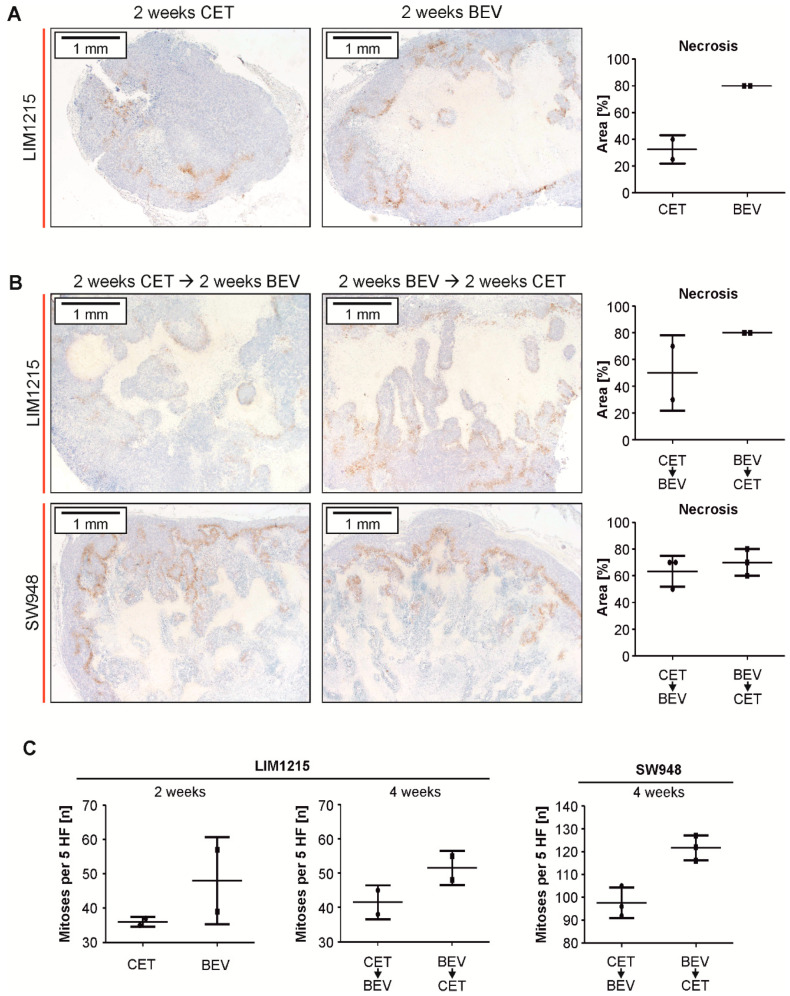
Analysis of experimental tumors after treatment with cetuximab (CET), bevacizumab (BEV), or both cetuximab and bevacizumab (CET->BEV and BEV->CET, respectively). (**A**) LIM1215 tumors were treated with either cetuximab or bevacizumab for two weeks and then stained for CAIX. Representative sections (left) and proportions of necrosis (right, *n* = 2) are depicted (formally *p* < 0.05, student’s t-test). (**B**) LIM1215 (upper panel) and SW948 (lower panel) tumors were sequentially treated as indicated for four weeks and then stained for CAIX. Representative sections (left) and proportions of necrosis (right, LIM1215: *n* = 2, SW948: *n* = 3) are depicted (formally *p* = not significant, student’s t-test). (**C**) The number of mitotic figures was counted in five consecutive high-power fields (LIM1215: *n* = 2, *p* = not significant, SW948: *n* = 3, *p* < 0.01, Student’s t-test).

In order to estimate the extent of pronounced hypoxia, we collected tissue samples of an exploratory cohort of pre-defined subcutaneous tumors (*n* = 2 per group for LIM1215 tumors and *n* = 3 per group for SW948 tumors). In LIM1215 tumors, treatment with bevacizumab for two weeks resulted in a trend toward widespread necrosis and induction of carbonic anhydrase (CA) IX as opposed to treatment with cetuximab (Figure 6A) or vehicle (Figure S5). In contrast, after sequential treatment, the extent of necrosis was comparable between the two sequence groups (Figure 6B) and increased in comparison to the control tumors in the SW948 model (Figure S5). The number of mitotic figures seemed to be lower in LIM1215 tumors that had been treated with cetuximab alone (CET) or first (CET->BEV) (Figure 6C). In SW948 tumors, this effect was statistically significant (Figure 6C). Notably, sequential treatment did not alter levels of EGFR in experimental tumors (Figure S6).

## 3. Discussion

The aggregated data from randomized trials indicate (i) that a sequence of bevacizumab plus chemotherapy in first-line followed by cetuximab plus chemotherapy in second-line is inferior to the inverse sequence with cetuximab plus chemotherapy as the first-line therapy and bevacizumab plus chemotherapy as the second-line treatment in *RAS* wildtype mCRC and (ii) that combination therapy with bevacizumab and anti-EGFR antibodies in the first-line setting has underadditive or even antagonistic, detrimental effects [7,8,10]. Some mechanisms underlying these findings have been proposed including an impaired distribution of the EGFR antibody or an upregulation of survival signals [21,22], but none have been rigorously evaluated.

Our data provide additional evidence for the inferior efficacy of the bevacizumab-cetuximab sequence: cetuximab protects CRC cells from a bevacizumab-mediated therapeutic deprivation of nutrients and oxygen (Figure 2) [23,24]. We have previously shown that EGFR inhibition protects human glioblastoma cells from oxygen and nutrient deprivation [16]. Applying these findings to CRC cells demonstrates that this is a robust effect. Accordingly, protection from hypoxia-induced cell death was also independent from the employed inhibitor (Figure 2B,C). Mechanistically, EGFR inhibition causes a preservation of cellular energy and mitochondrial stabilization [16] and cells defective in inhibiting EGFR-dependent downstream signaling pathways in response to deprivation are, in contrast, sensitized to hypoxia-induced cell death [25]. Furthermore, cetuximab has been shown to downregulate HIF-1α and thereby lactate dehydrogenase (LDH)-A, resulting in an inhibition of glucose consumption, lactate production, and intracellular ATP levels [26]. It is noteworthy that at least some EGFR inhibitors exert cytotoxicity preferentially under nutrient replete conditions [17]. Novel approaches even aim to improve tumor oxygenation concurrent to treatment [27] because hypoxia may promote tumor progression and resistance to current therapeutic strategies [28,29].

The occurrence of hypoxia is an important characteristic of the growth of solid tumors and contributes significantly to the response or resistance of a tumor to therapy. It should be noted that a solid tumor may simultaneously have regions of moderate (O_2_ ≤ 2%) and severe hypoxia (O_2_ ≤ 1%) [30] as well as acute and chronic hypoxia [31]. Many changes in the tumor microenvironment are induced by acute hypoxia and mediated by the stabilization and induction of HIF-1 and HIF-2. Chronic hypoxia tends to destabilize HIF-1 and HIF-2 [32], while in in vitro experiments, a more acute hypoxia setting was used, and the in vivo experiments with rapid tumor growth with necroses included elements of a state of chronic hypoxia. Bevacizumab is also known to induce both acute hypoxia in [33] and chronic hypoxia due to reduced vascular sprouting [34].

The upregulation of VEGF in the course of resistance to cetuximab is an additional reason for saving bevacizumab for second-line therapy [11,12]. It appears plausible that our findings might be relevant in other tumor entities where antiangiogenic and anti-EGFR strategies are therapeutic options (e.g., the EGFR inhibitor lapatinib was investigated in combination with bevacizumab in breast cancer [35]), however to our knowledge, no results of two-arm comparative trials have been reported as yet.

A limitation of our experimental design is that a predefined sequential treatment with VEGF and EGFR antibodies (without a chemotherapy backbone) is not a therapeutic standard for mCRC patients. This treatment course was chosen to specifically investigate whether one antibody affects the treatment efficacy of the other antibody. As expected, the application of bevacizumab enhanced necrosis and CAIX protein in LIM1215 xenografts, suggesting hypoxic conditions (Figure 6A). Cetuximab as well as the CET->BEV sequence might better suppress the mitotic activity than bevacizumab (Figure 6C). This aspect needs to be verified in larger cohorts, but is in accordance with the survival of mice of the corresponding groups (Figure 4 and Figure 5). To account for the *TP53* spectrum in colorectal cancer, we used both *TP53* wildtype cells (LIM1215) as well as *TP53* mutant cells (SW948 *TP53* deletion frameshift) [36]. Wildtype *TP53* status has been associated with improved survival in rectal cancer and colorectal when the chemotherapy protocol included cetuximab [37,38]. In bevacizumab treated colorectal cancer patients, *TP53* gain of function mutations have been associated with improved [39] and *TP53* truncating mutations with reduced survival of patients [40]. Mutations of the tumor suppressor transcription factor *TP53* as well as of the oncogenic kinase *RAS* have been shown to synergistically induce genetic programs that drive transformation [41,42]. While we did not include pairs of *TP53* functional and non-functional sub cell lines in our experiments, the observed effects of protection against hypoxia-induced cell death were present, regardless of *TP53* status.

In our experimental setup, the in vivo efficacy of the CET->BEV sequence as well as the in vitro hypoxia-protective effect of EGFR inhibition were detectable regardless of cellular *KRAS* status, indicating a potentially unrecognized efficacy of sequential therapy even in *KRAS* mutant mCRCs. In line with our observations, Taniguchi et al. recently reported that sequential administration of panitumumab followed by bevacizumab in subcutaneous LIM1215 xenografts reduced tumor growth in comparison to vehicle treatment while the reverse sequence did not [43]. However, the absolute values of difference were very minor and the applied experimental settings differed from ours with a much smaller tumor size at the baseline. Data from xenograft experiments with colorectal cancer cell lines SW48 (*RAS* wildtype), HT-29 (*RAS* mutant), and SW620 (*RAS* mutant) presented by Mésange et al. indicated a superiority of a combined exposure to bevacizumab and erlotinib, an inhibitor of the EGFR tyrosine kinase, in comparison to bevacizumab alone [44]. The approach of the related clinical trial GERCOR DREAM [45] differed from ours in that all patients had been treated with bevacizumab before randomization to treatment with bevacizumab alone or with a combination of bevacizumab and erlotinib. We did not test concomitant treatment with bevacizumab and cetuximab because of the aforementioned discouraging results from clinical trials [7,8]. In their retrospective analysis, Derangère et al. observed that a previous anti-VEGF therapy decreased anti-EGFR efficacy in *KRAS* and *NRAS* wildtype CRC, resulting in shorter PFS [22]. Their in vitro experiments with cell lines Colo320, SW48, and Caco-2 revealed a cytotoxic effect of cetuximab; here, cell death was analyzed 48 h following treatment with cetuximab in a concentration of 500 µg/mL. Pre-incubation with VEGF-A reduced cell death associated with cetuximab treatment. As an increase in VEGF-A plasma levels after administration of bevacizumab has been suggested by a pharmacokinetic model [46], this mitigation might contribute to a negative impact of bevacizumab on subsequent anti-EGFR therapy.

## 4. Materials and Methods

### 4.1. Cell Lines

The human colon carcinoma cell lines LIM1215 (*KRAS* wildtype, RRID:CVCL_2574) and SW948 (*KRAS* mutant, RRID:CVCL_0632) [36,47,48] were kindly provided by Dr. J. Gebert (Heidelberg, Germany) and the human glioblastoma cell line LNT-229 (*KRAS* wildtype, RRID:CVCL_0393) by Dr. N. de Tribolet (Lausanne, Switzerland) [25]. The mutational profiles of the employed cell lines are summarized below (Table 1) (Cancer Cell Line Encyclopedia, https://portals.broadinstitute.org/ccl [49,50]). All cells were maintained in Dulbecco’s Modified Eagle’s Medium (DMEM) containing 10% fetal calf serum (FCS), 2 mM glutamine, 100 IU/mL penicillin, and 100 µg/mL streptomycin (Life Technologies, Carlsbad, CA, USA).

### 4.2. Generation of EGFR Gene-Suppressed Cells

The pLKO.1 plasmids targeting the *EGFR* (EGFRsh) and the pLKO.1 plasmid with a non-targeting shRNA sequence (NTsh) were ordered from Sigma-Aldrich (St. Louis, MO, USA, TRCN0000121068, TRCN0000295969) and Addgene (Cambridge, MA, USA, #1864). Lentivirus was produced according to the Addgene protocol in HEK293 cells with the packaging plasmid pCMV-dR8.2 dvpr (Addgene #8455) and the envelope plasmid pCMV-VSVG (Addgene #8454). Polybrene (Millipore, Burlington, MA, USA) was used to facilitate transduction. For selection of transduced cells, 2 µg/mL puromycin (Sigma, Taufkirchen, Germany) was added to the culture medium.

### 4.3. Reagents

Cetuximab was provided as a stock solution (5 mg/mL) by Merck (Darmstadt, Germany). The EGFR inhibitors Erlotinib and PD153035 were purchased from Sigma (Taufkirchen, Germany). Bevacizumab stock solution (25 mg/mL) was obtained from Roche (Mannheim, Germany) via the pharmacy of the University Hospital Frankfurt.

### 4.4. Induction of Hypoxia

Hypoxia was induced as previously described by incubation in Gas Pak pouches for anaerobic culture (Becton-Dickinson, Heidelberg, Germany) [19]. Cells were seeded in DMEM containing 10% FCS and allowed to attach overnight. Subsequently, the medium was removed and the cells were incubated in serum-free DMEM adjusted to 2 mM glucose under normoxia (21% oxygen) or hypoxia (0.1% oxygen).

### 4.5. Cell Density and Cell Viability Assays

Cell density was assessed by crystal violet (CV) staining [51]. Viability was determined by propidium iodide (PI) uptake and flow cytometry (BD Canto II, Heidelberg, Germany, and Summit 4.2. software) [19]. Release of lactate dehydrogenase (LDH) was measured using the Cytotoxicity Detection Kit (LDH) (Roche, Mannheim, Germany) [19]. LDH release was calculated with reference to the maximum, established by cell lysis with 1% Triton X-100, in every condition (e.g., for both NTsh and EGFRsh cells in both normoxia and hypoxia).

### 4.6. RNA Extraction and Quantitative Reverse Transcription-PCR Analysis

Quantitative reverse transcription-PCR (qPCR) was performed using a standard protocol as described by Wanka et al. [52]. Briefly, RNA was purified using TRIzol (Invitrogen, Carlsbad, CA, USA) and the RNeasy Kit (Qiagen, Hilden, Germany) was used. cDNA was synthesized by applying SuperScript VILO (Invitrogen, 10 min at 25 °C followed by 2 h at 42 °C). The reaction was stopped at 85 °C for 10 min. qPCR was realized in an iQ5 real-time PCR detection system (Bio-Rad, Hercules, CA, USA) using the ABsolute Blue qPCR SYBR-Green Fluorescein Mix (Thermo Fisher Scientific, Waltham, MA, USA) and the corresponding primer pairs (18S rRNA and succinate-dehydrogenase complex subunit A (SDHA) were used as housekeeping genes for normalization) (Table 2).

### 4.7. Immunoblot Analysis

After the incubation period, cells were washed with ice-cold phosphate buffered saline (PBS) and immediately frozen by placing the dishes in fluid nitrogen. Lysates were prepared as described previously using lysis buffer P and subjected to SDS-PAGE [19]. Membranes were probed with antibodies directed against actin (Santa Cruz Biotechnology, Santa Cruz, CA, USA), EGFR (Santa Cruz Biotechnology), HIF-1α (BD Transduction Laboratories, San Jose, CA, USA), BNIP3 (Abcam, Cambridge, UK), and phosphorylated (P-) p90RSK, P-Akt, P-p42/44 MAP kinase (MAPK), P-ribosomal protein S6 (P-RPS6) as well as the eukaryotic translation initiation factor 4E (eIF4E9) as a loading control using the PathScan Multiplex Western Cocktail I (Cell Signaling Technology, Danvers, MA, USA, #7100). The secondary anti-rabbit and anti-mouse antibodies were purchased from Santa Cruz Biotechnology. Chemiluminescence solution was used for detection [25].

### 4.8. Animal Experiment, Tumor Histology, and Immunohistochemistry

Animal experiments were approved by the local authority (Regierungspräsidium, Darmstadt, Germany) and performed in accordance with the ARRIVE-guidelines and institutional guidelines [53]. Sample size was calculated using the resource equation approach (Sample Size Calculations (IACUC)) [54,55] For the generation of subcutaneous tumors, 4 × 10^6^ tumor cells in 100 μL PBS were injected subcutaneously in the lower back of 6-week-old female athymic nude mice (Foxn1nu, Harlan, Indianapolis, IN, USA). Tumor size was evaluated twice weekly using a manual caliper. Tumor volume was calculated using the formula height * height * width/2. The tumors were allowed to grow to a volume of 500 mm^3^ before treatment was initiated. Treatment consisted of an intraperitoneal injection of 30 µg/g cetuximab or PBS once weekly and of 10 µg/g bevacizumab or PBS twice weekly (in accordance with previously published effective doses) [56,57]. After two weeks, treatment was switched in the “sequence groups” without any treatment pause and stopped in the “monotherapy groups”. Mice were sacrificed when the tumor reached a volume of 1500 mm^3^ or tumor ulceration was detectable. Staining for carbonic anhydrase (CA) IX, (Cell Signaling Technology, Danvers, MA, USA) and EGFR (Santa Cruz Biotechnology, Santa Cruz, CA, USA) was performed on slides from formalin-fixed paraffin-embedded tumor specimens following a standard protocol and evaluated by an experienced pathologist (M.M. and P.N.H.).

### 4.9. Statistics

For survival analysis, a Gehan–Breslow–Wilcoxon test was applied using GraphPad Prism version 8 (GraphPad Software, La Jolla, CA, USA). The Student’s t-test was calculated using Excel (Microsoft, Seattle, WA, USA).

### 4.10. Ethics Approval

This work was approved by the Ethics Committee of Frankfurt University (Ethik-Kommission, University Hospital Frankfurt, Goethe University) with the reference number SNO_04-09.

## 5. Conclusions

Our study addressed a frequently used therapy sequence of two antibodies and revealed that cetuximab could mediate protection against hypoxia-induced cell death potentially caused by bevacizumab. This finding constitutes a mechanistic rationale to rely on cetuximab prior to bevacizumab in the treatment of *RAS* wildtype left sided colorectal cancer to optimize therapy.

## Figures and Tables

**Table 1:** Mutational status of KRAS, TP53, phosphatase and tensin homolog (PTEN), epidermal growth factor receptor (EGFR), and retinoblastoma (RB) genes (Cancer Cell Line Encyclopedia, https://portals.broadinstitute.org/ccle [49,50]).

	*KRAS*	*TP53*	*PTEN*	*EGFR*	*RB*
LIM1215	wildtype	wildtype	wildtype	wildtype	wildtype
SW948	mutant	mutant	wildtype	wildtype	wildtype
LNT-229	wildtype	wildtype	wildtype	wildtype	wildtype

**Table 2:** Primer pairs used for qPCR. Primer pairs for detection of 18S rRNA, succinate-dehydrogenase complex subunit A (SDHA) and epidermal growth factor receptor (EGFR).

Gene	Forward Primer (5′-3′)	Reverse Primer (5′-3′)
18S	CGGCTACCACATCCAAGGAA	GCTGGAATTACCGCGGCT
SDHA	TGGGAACAAGAGGGCATCTG	CCACCACTGCATCAAATTCATG
EGFR	GCGTTCGGCACGGTGTATAA	GGCTTTCGGAGATGTTGCTTC

## Supplementary Materials

The following are available online at https://www.mdpi.com/2072-6694/12/10/3050/s1, Figure S1: Effects of EGFR inhibition on signal transduction in colon cancer cell lines, Figure S2: Cetuximab-mediated protection from hypoxia-induced cell death in colon cancer cell lines, Figure S3: Effects of cetuximab and bevacizumab on hypoxia-induced cell death in colon cancer cells with EGFR gene suppression, Figure S4: Growth curves of subcutaneous tumor xenografts, Figure S5: Comparison of necroses extent and mitoses frequency with control tumors, Figure S6: Immunohistochemistry staining for EGFR in representative experimental tumors, Figure S7: Uncropped immunoblot figures, Figure S8: Uncropped immunoblot figure. Immunoblot figure of Figure S1.
