# Supplementary material for: Cetuximab-Mediated Protection from Hypoxia- Induced Cell Death: Implications for Therapy Sequence in Colorectal Cancer
Source: Cancers (Basel). 2020 Oct 20;12(10):3050. doi: 10.3390/cancers12103050 (PMC7589936; doi:10.3390/cancers12103050)
Supplement: Supplementary file 1 [file cancers-12-03050-s001.pdf]

Supplementary Materials: Cetuximab-Mediated Protection From Hypoxia- Induced Cell Death: Implications for Therapy Sequence in Colorectal Cancer

Hans Urban, Gabriele D. Maurer, Anna-Luisa Luger, Nadja I. Lorenz, Benedikt Sauer, Christopher Stroh, Jörg Trojan, Michel Mittelbronn, Joachim P. Steinbach, Patrick N. Harter and Michael W. Ronellenfitsch

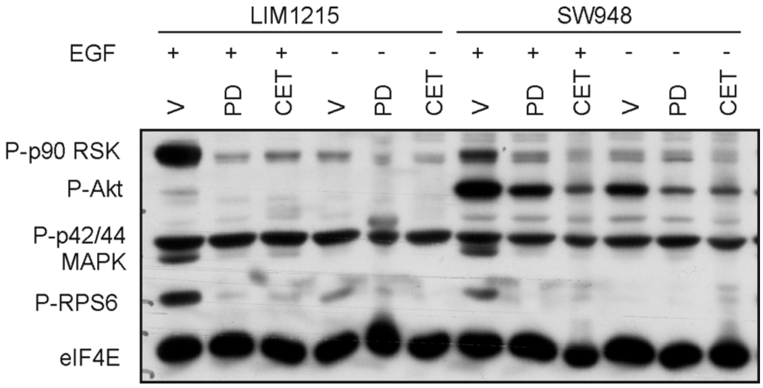

Figure S1. Effects of EGFR inhibition on signal transduction in colon cancer cell lines. LIM1215 and SW948 cells were stimulated with 10 ng/ml EGF or DMSO vehicle for 30 min and in the presence of vehicle (V), 10 μM PD153035 (PD) or 100 μg/ml cetuximab (CET). Lysates were then analyzed for phosphorylated (P-) p90RSK, P-Akt, P-p42/44 MAPK, P-RPS6 and, as a loading control, eIF4E. Uncropped Immunoblot Figure in Figure S8.

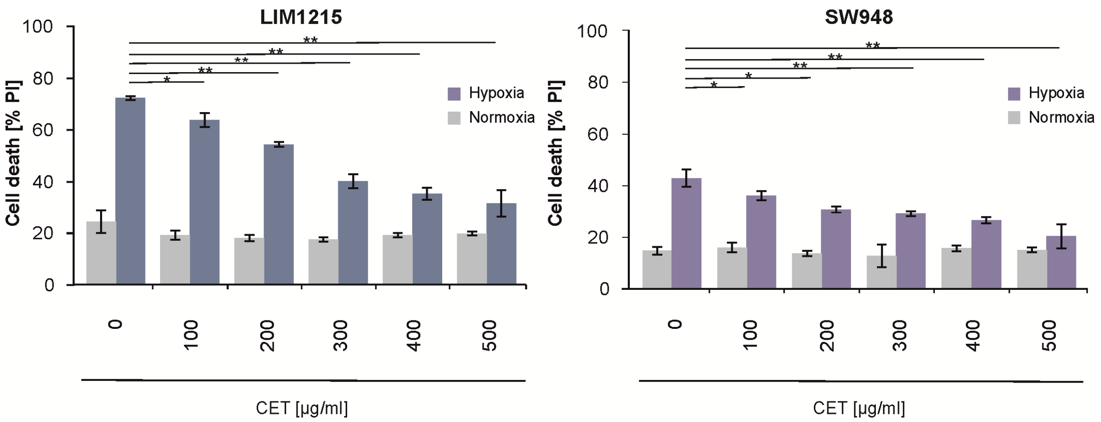

Figure S2. Cetuximab-mediated protection from hypoxia-induced cell death in colon cancer cell lines. LIM1215 and SW948 cells were treated with vehicle or cetuximab (CET) at concentrations ranging from 100 to 500 μg/mL for 24 h. Then cell death was assessed by PI staining and flow cytometry (n = 3, mean ± SD, * p < 0,05 ** p < 0.01).

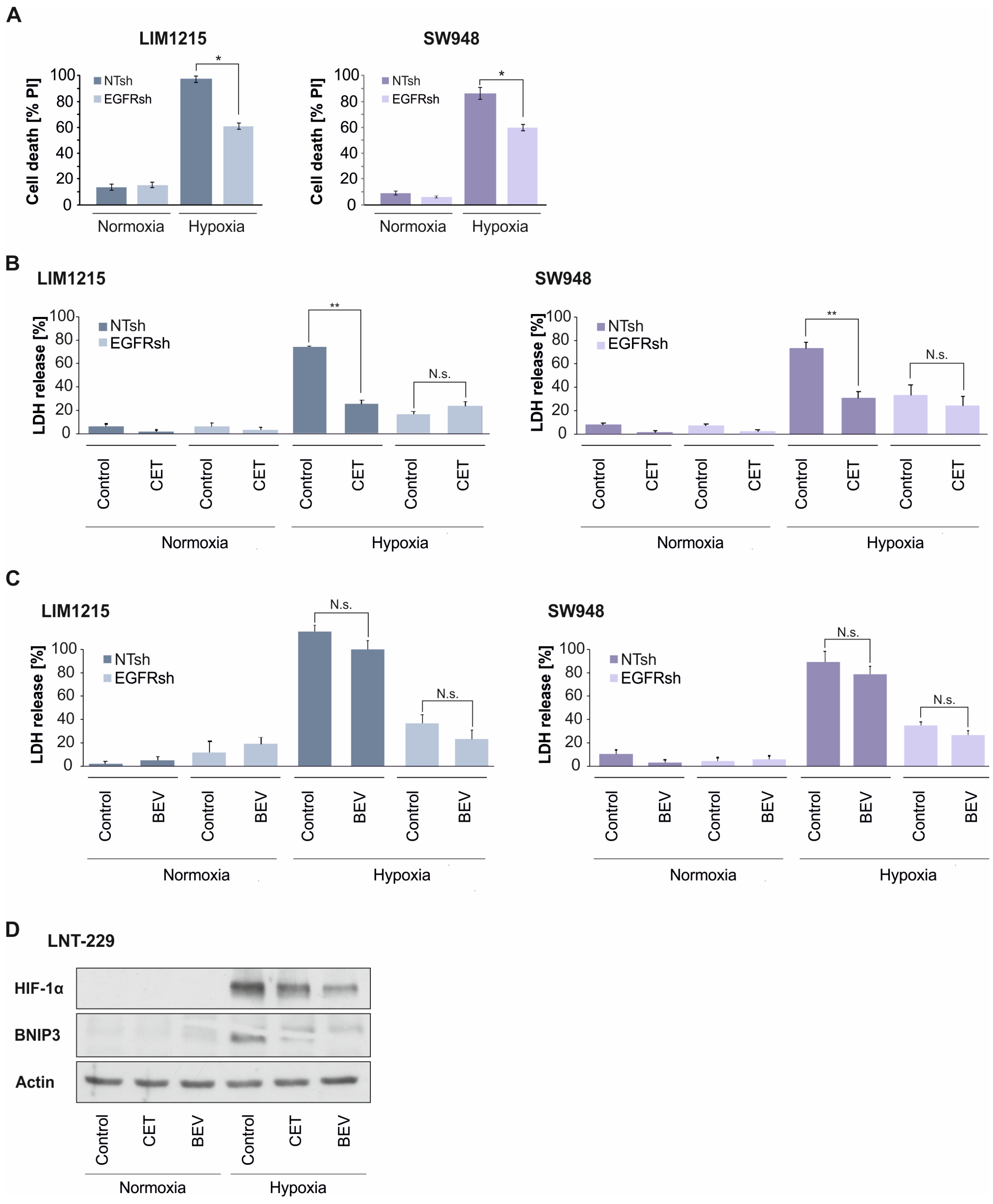
A
LIM1215
SW948
Cell death [% PI]
NTsh
EGFRsh
*
Normoxia
Hypoxia
B
LIM1215
SW948
LDH release [%]
NTsh
EGFRsh
**
N.s.
Control
CET
Normoxia
Hypoxia
C
LIM1215
SW948
LDH release [%]
NTsh
EGFRsh
N.s.
Control
BEV
Normoxia
Hypoxia
D
LNT-229
HIF-1α
BNIP3
Actin
Control
CET
BEV
Normoxia
Hypoxia

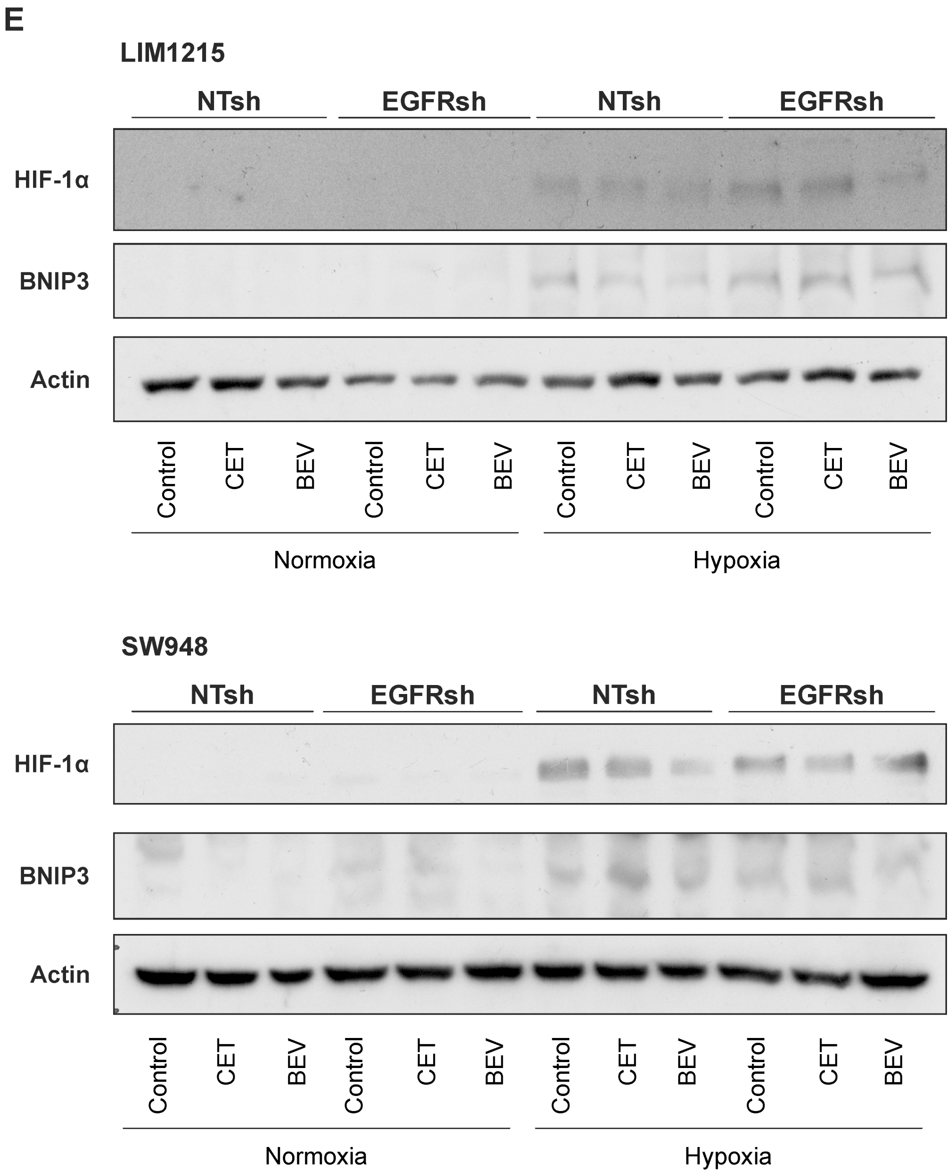

Figure S3. Effects of cetuximab and bevacizumab on hypoxia-induced cell death in colon cancer cells with EGFR gene suppression. (**A**) EGFR-gene suppression protects colon cancer cell lines from hypoxia-induced cell death. LIM1215 and SW948 NTsh and EGFRsh (Seq. 1) cells were cultured in serum-free DMEM containing 2 mM glucose and subjected to normoxic and hypoxic conditions, respectively. 24 h later, cell viability was evaluated by PI staining and flow cytometry (mean ± SD; * $p < 0.05$, ** $p < 0.01$). (**B**) NTsh and EGFRsh LIM1215 and SW948 cells were treated with vehicle or 300 μg/ml cetuximab (CET) for 24 h. Cell death was measured by LDH release ($n = 4$, mean ± SD; N.s. = not significant, ** $p < 0.01$). (**C**) NTsh and EGFRsh LIM1215 and SW948 cells were treated with vehicle or 300 μg/ml bevacizumab (BEV) for 24 h. Cell death was measured by LDH release ($n = 4$, mean ± SD; N.s. = not significant). (**D**) LNT-229 cells were incubated for 8 h under normoxia or hypoxia in serum-free medium with 2 mM glucose in the presence of 300μg/mL cetuximab (CET) or bevacizumab (BEV). Cellular lysates were analyzed by immunoblot with antibodies for HIF-1α, BNIP3 (please note the small tear in the gel) or actin. (**E**) LIM1215 (upper panel) and SW948 (lower panel) NTsh and EGFRsh cells were incubated and analyzed as in (**D**).

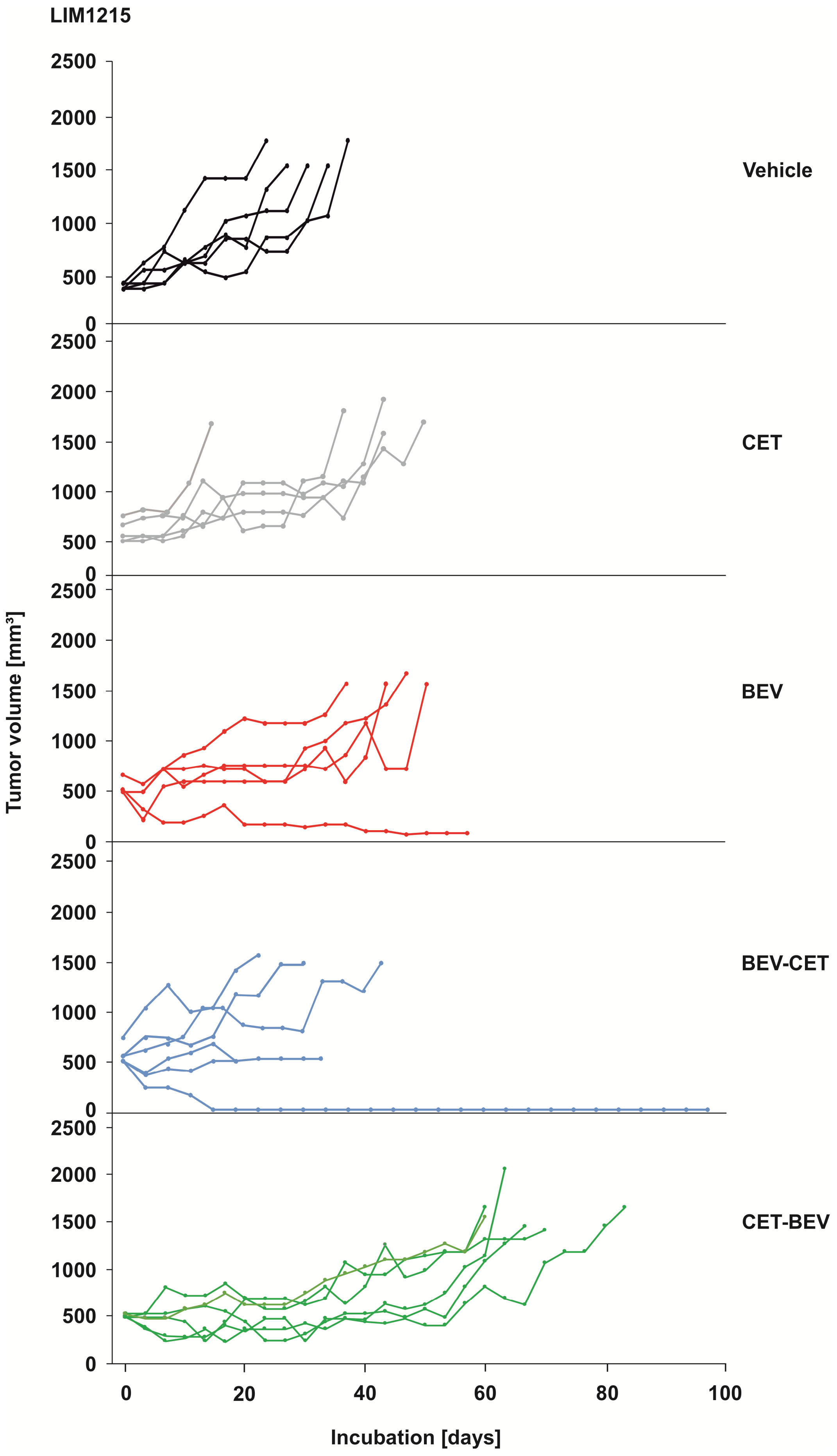
LIM1215
Tumor volume [mm³]
2500
2000
1500
1000
500
0
Vehicle
CET
BEV
BEV-CET
CET-BEV
0
20
40
60
80
100
Incubation [days]

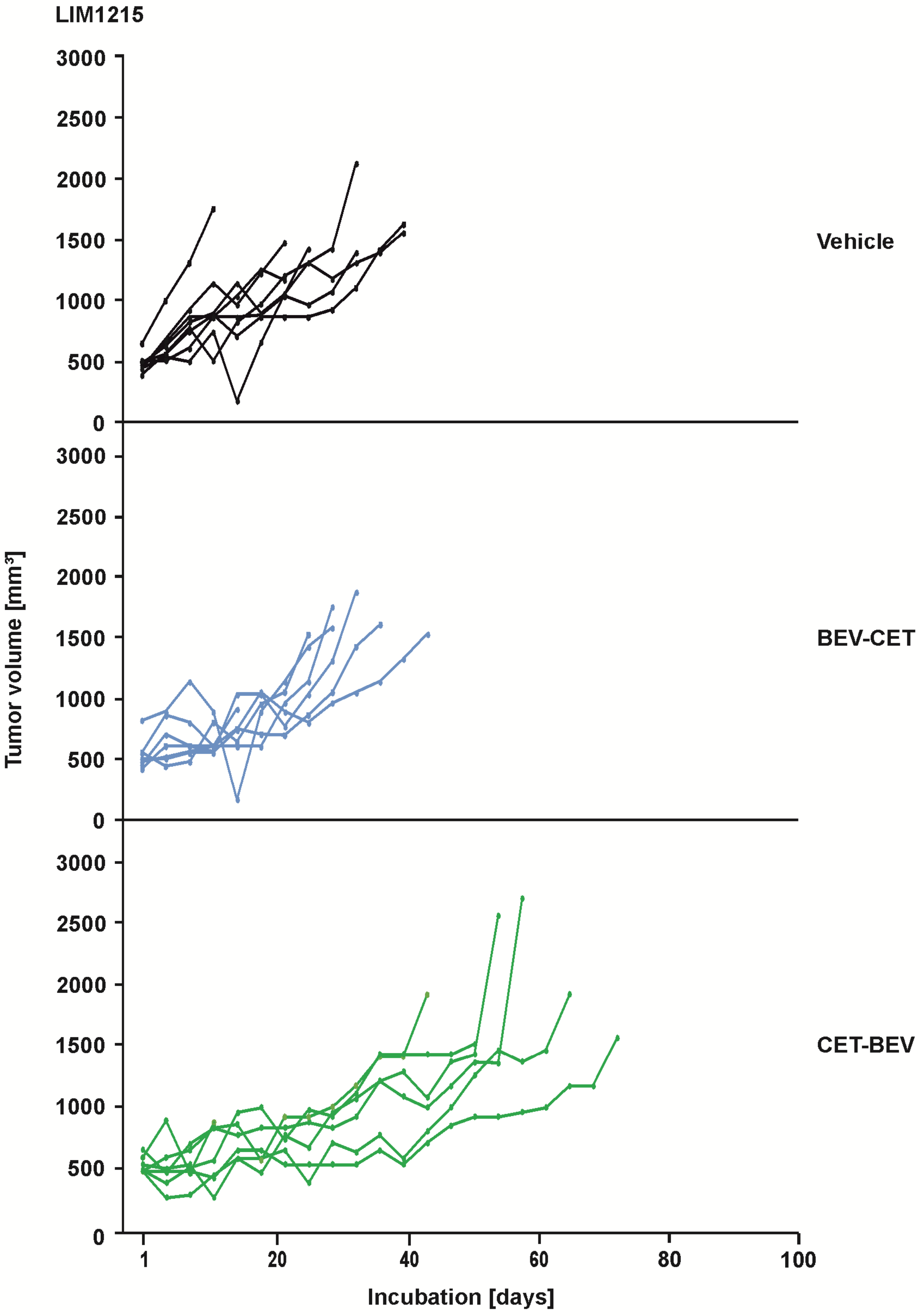
LIM1215
Vehicle
BEV-CET
CET-BEV
Tumor volume [mm³]
Incubation [days]
3000
2500
2000
1500
1000
500
0
1
20
40
60
80
100

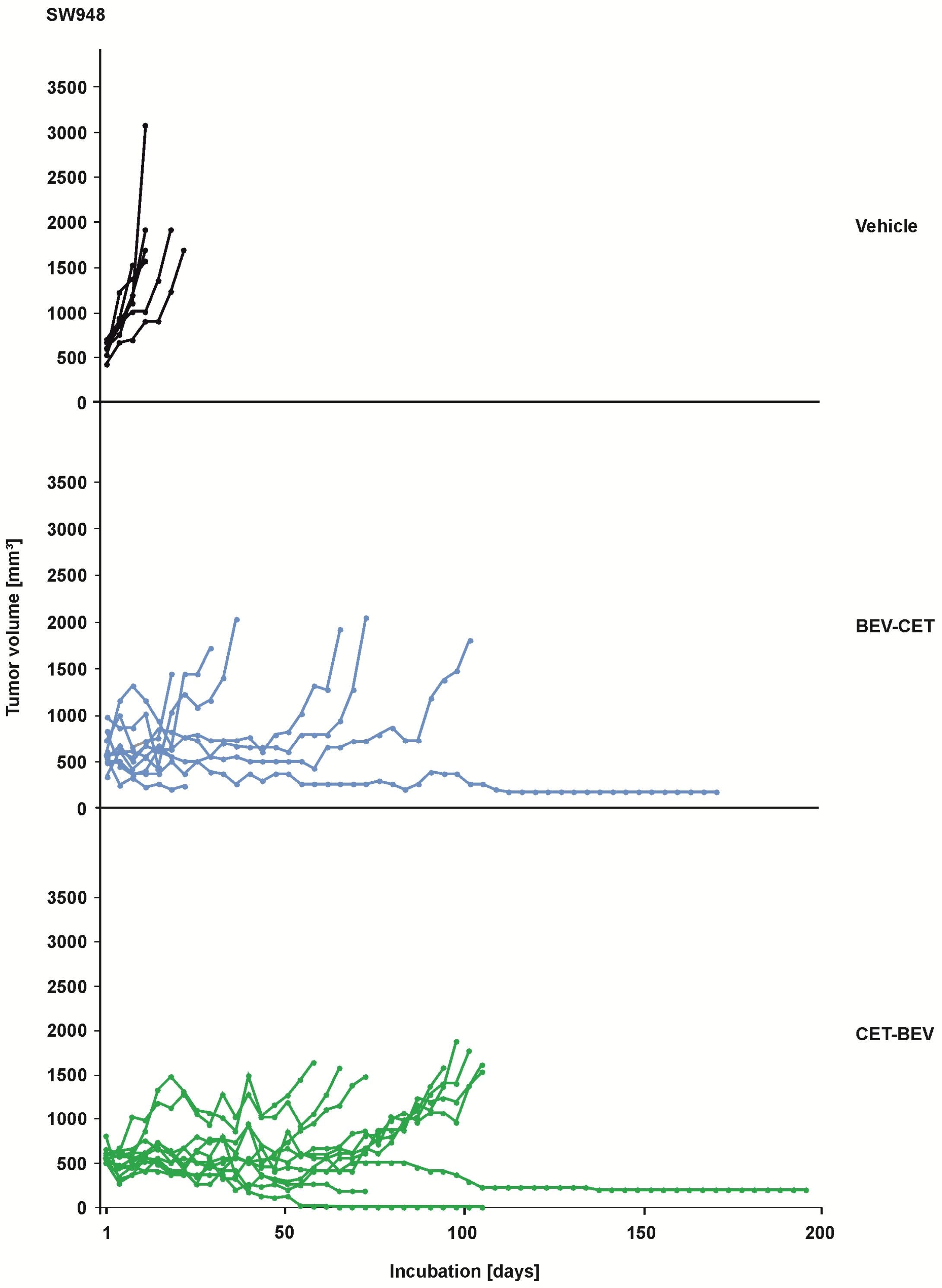

Figure S4. Growth curves of subcutaneous tumor xenografts (**A**) Subcutaneous LIM1215 tumor volumes for each individual animal, treated as indicated in Fig. 4, are depicted over time. (**B,C**) Subcutaneous LIM1215 and SW948 tumor volumes for each individual animal, treated as indicated in Fig. 5, are depicted over time.

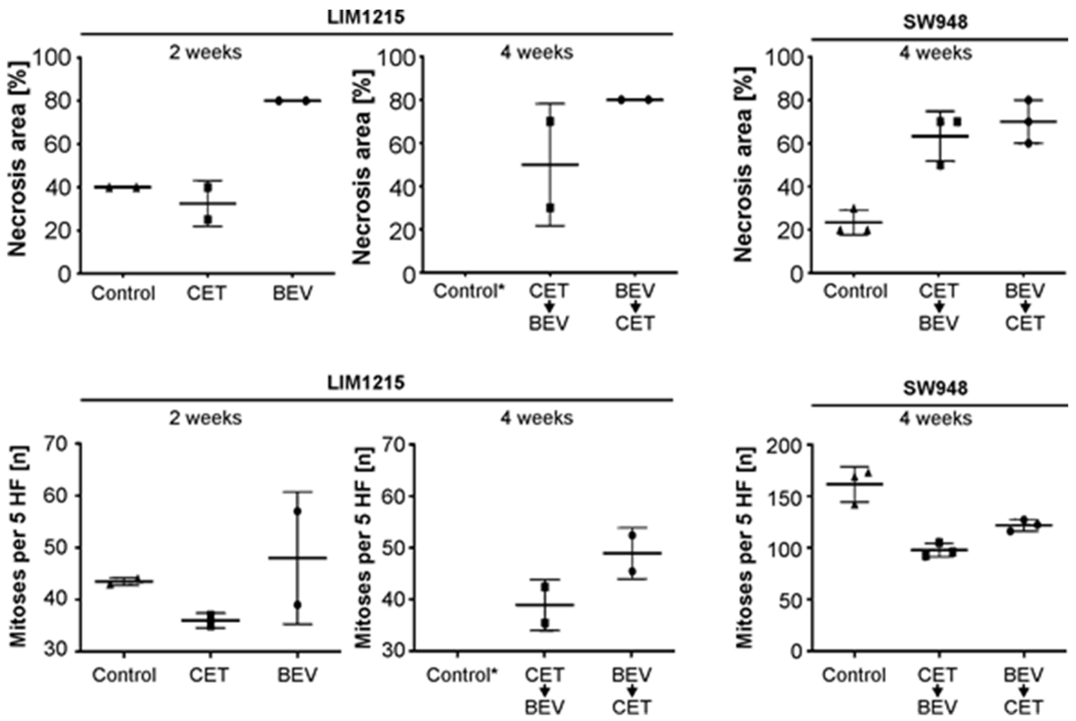

Figure S5. Comparison of necroses extent and mitoses frequency with control tumors. Necrosis area (upper panel) and mitoses frequency (lower panel) of CET->BEV and BEV->CET treated experimental tumors (as also depicted in Figure. 6) were compared with the corresponding control tumors (* no meaningful analysis possible).

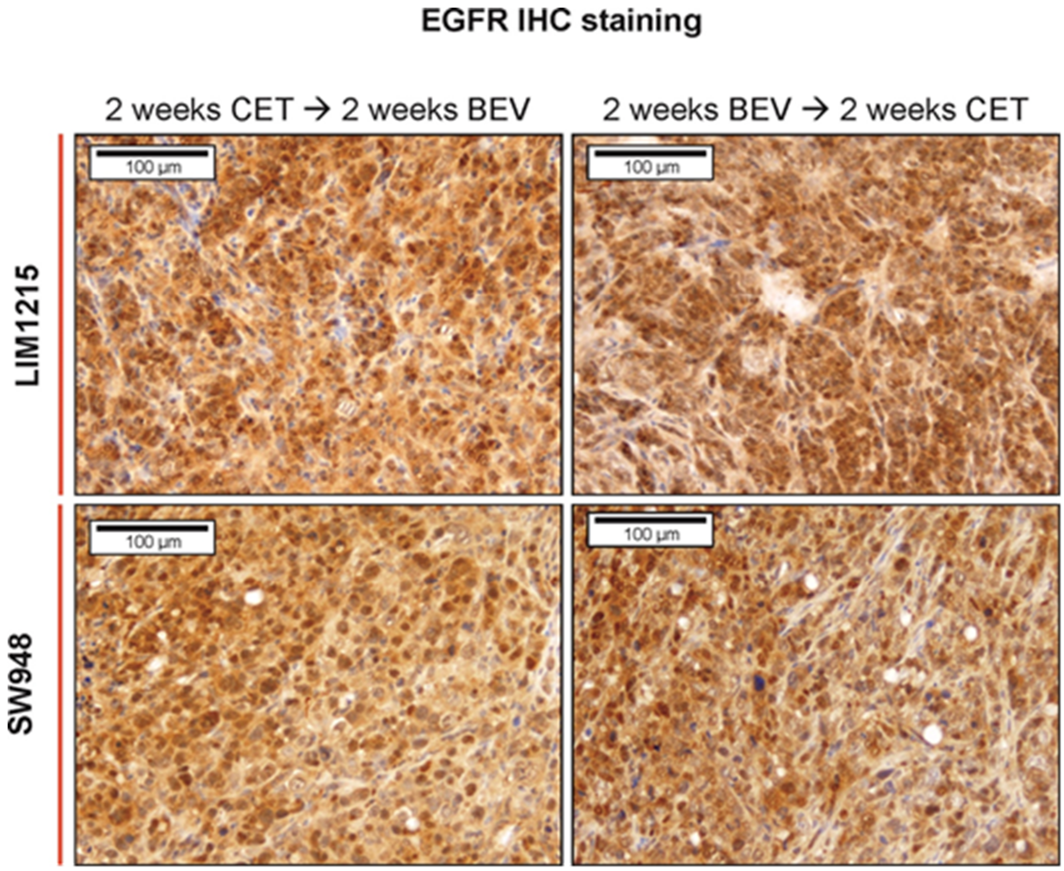

Figure S6. Immunohistochemistry staining for EGFR in representative experimental tumors. Tumor sections were analyzed for expression of EGFR.

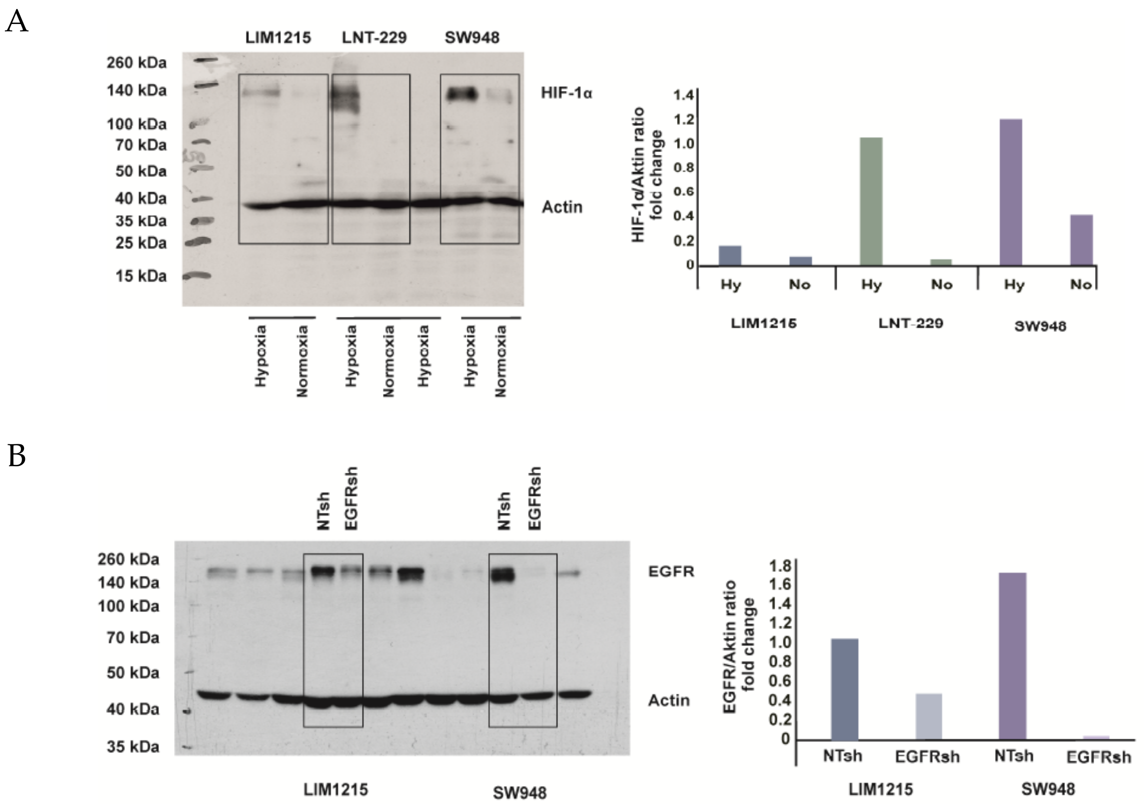

Figure S7. Uncropped western blot figures. (**A**) Immunoblot figure of Figure 2. (**B**) Immunoblot figure of Figure 3.

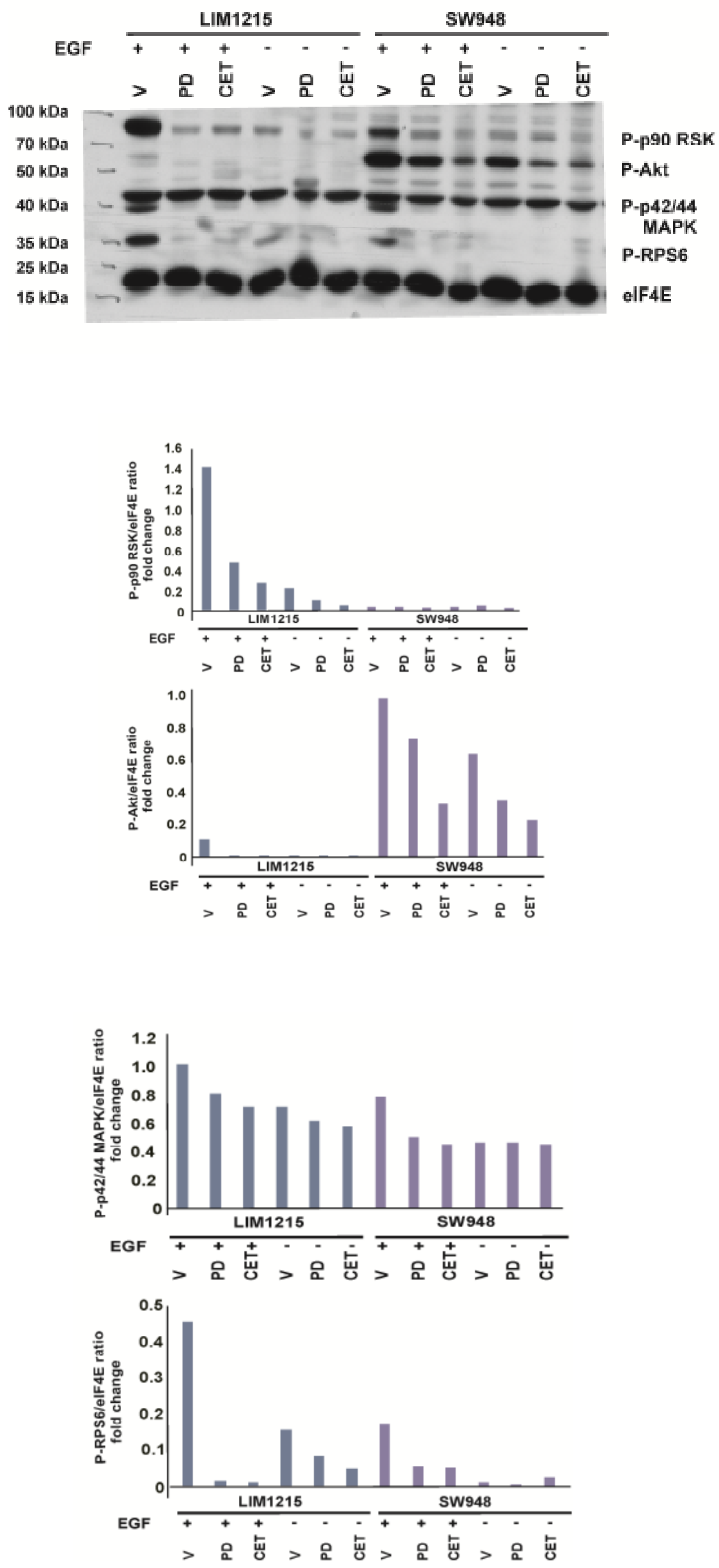

Figure S8. Uncropped western blot figure. Immunoblot figure of Figure S1.

© 2020 by the authors. Submitted for possible open access publication under the terms and conditions of the Creative Commons Attribution (CC BY) license (http://creativecommons.org/licenses/by/4.0/).
